# Foreign Body Stocked between Vocal Cords

**Published:** 2016

**Authors:** Kamran Mottaghi, Farhad Safari, Masoud Nashibi

**Affiliations:** Department of Anesthesiology, Shahid Beheshti University of Medical Sciences, Tehran, Iran

**Keywords:** Foreign Body, Vocal Cords, Anesthesia management

## Abstract

Foreign body (FB) aspiration is a rare event in adults and can mimic other pathologies such as refractory asthma. Most of the objects can be removed using a bronchoscope. Herein, we present a unique case of aspiration of a FB stocked between vocal cords for one week and the anesthetic considerations.

## INTRODUCTION

Foreign body aspiration, although common in children, is a rare event in adults (1, 2) as adults comprise only 20% of aspiration cases, mostly in the sixth or seventh decade of life (3). Aspirated FB may remain unrecognized for months (4, 5). The most common objects encountered as FBs in adults are food particles and broken parts of denture (1). In general, 27% of cases in adults are due to dental prosthesis aspiration (4), mostly occurring during sleep (2, 4) or dental treatment (6, 7). Bronchoscopy is the preferred method for removal of FBs (4, 7) which is done under general anesthesia, sedation or local anesthesia (2, 4, 6, 7). Herein, we report a male patient who aspirated his dental prosthesis and it was lodged between vocal cords.

## CASE SUMMARIES

A 27-year-old male presented to an otorhinolaryngology clinic complaining of dyspnea and low pitched voice. His problem had begun one week earlier while he was asleep. He mentioned a feeling of suffocation and FB in his pharynx. He started to cough vigorously and lost her ability to talk normally. During the previous week, he had a couple of visits to different physicians with no definitive diagnosis. He had no past medical or surgical history with normal vital signs. During indirect laryngoscopy, a dental prosthesis was noticed between the vocal cords by a junior resident; thus, the patient was scheduled for direct laryngoscopy under general anesthesia in the operating room. In the operating room, the patient was calm, with stable vital signs and normal physical examination and normal respiratory sounds.

**Figure 1.**
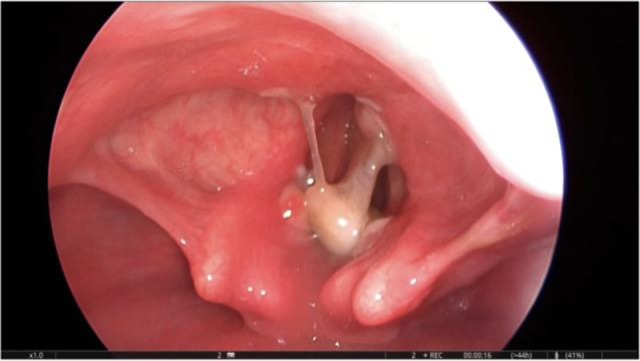
Foreign body stocked between vocal cords

**Figure 2.**
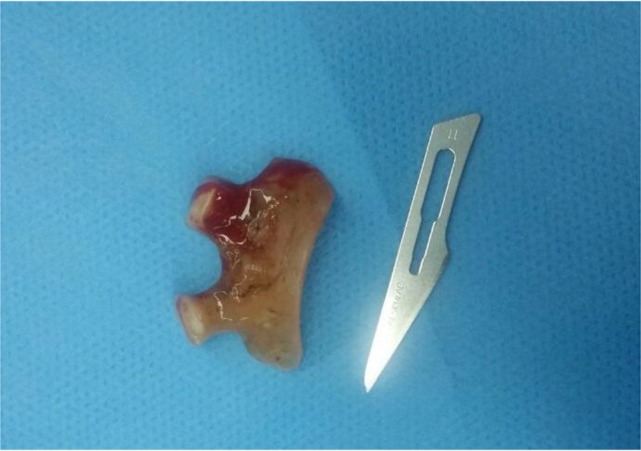
Foreign body

We chose the inhalation induction using increasing percentages of sevoflurane to maintain spontaneous ventilation. When the depth of anesthesia was acceptable (cerebral state index 40), direct laryngoscopy was performed and dental prosthesis was detected between vocal cords (Figure 1). After spraying 3 puffs of 10% lidocaine on vocal cords, ENT surgeon gently dislodged and removed the dental prosthesis (Figure 2). On the day after the procedure, the patient left the hospital with no respiratory problem.

## DISCUSSION

Foreign body aspiration occasionally occurs in adults (1, 2) unless some predisposing factors are present such as stroke, encephalopathy, alcohol abuse, seizure, sedative and illicit drug abuse, facial trauma, mental retardation (4, 8) and dementia (2). Swallow and cough reflex are efficient defense lines against aspiration which put the patients at risk when surpassed (1). Aspiration may occur in otherwise normal individuals during loud speaking, laughing or shouting (6). The most common presenting symptoms are dyspnea, chronic cough and hemoptysis which could be misdiagnosed and treated as obstructive airway diseases such as asthma that are unresponsive to anti-inflammatory and bronchodilator medications (1). Thus, recurrent pneumonia with no precise etiology or newly appeared asthma in an otherwise healthy patient with no past medical history should raise suspicion about FB aspiration. Longtime FB lodgment in airways may lead to pneumonia, abscess formation and bronchiectasis. Granulation tissue formation around the FB may resemble bronchial carcinoma (4, 7).

There is no specific radiographic findings and in CT scan, FB may be seen as an intra-tracheal, bronchial or parenchymal mass (1). Bronchoscopy, rigid or flexible, has been considered as the gold standard for evaluation of cases with high likelihood of aspiration (4) with rigid bronchoscopy under general anesthesia as the preferred approach for children and rigid bronchoscopy under general anesthesia or flexible bronchoscopy under sedation or local anesthesia for adults (2, 7). Flexible bronchoscopy is less invasive than the rigid type but the low grasp of instruments used through flexible bronchoscope has the potential risk of more manipulation of FB (4). Although dental aspiration usually has a good prognosis, there are infrequent complications such as bleeding or perforation which are attributed to late diagnosis (7) and require multidisciplinary approach (9).

Anesthesia management during bronchoscopy for FB aspiration is a challenge for anesthesiologists. There is no consensus on the method of choice for induction and maintenance of anesthesia; however, spontaneous ventilation during induction of anesthesia is commonly practiced by anesthesiologists in children. Anesthesia maintenance can be achieved via spontaneous ventilation over bronchoscope for proximal objects and through positive pressure ventilation in case of distal FBs (10).

There are many case reports about FB aspiration into trachea, trachobroncheal tree or pharynx (3) but our case report is unique since the FB was lodged between vocal cords for one week. Our challenge was induction of anesthesia and its maintenance, as in this case, intravenous administration of hypnotics could jeopardize the condition from spontaneous ventilation to cannot intubate and cannot ventilate situation.

## CONCLUSION

Foreign body aspiration could be life threatening per se and airway management and method of ventilation may increase the risk. Although there is no consensus on the airway management and ventilation method for these cases, spontaneous ventilation during bronchoscopy is widely practiced by anesthesiologists and appears to be the rational choice.
